# Nano and Technological Frontiers as a Sustainable Platform for Postharvest Preservation of Berry Fruits

**DOI:** 10.3390/foods12173159

**Published:** 2023-08-23

**Authors:** Maricarmen Iñiguez-Moreno, Reyna Berenice González-González, Elda A. Flores-Contreras, Rafael G. Araújo, Wei Ning Chen, Mariel Alfaro-Ponce, Hafiz M. N. Iqbal, Elda M. Melchor-Martínez, Roberto Parra-Saldívar

**Affiliations:** 1 School of Engineering and Sciences, Tecnologico de Monterrey, Monterrey 64849, Mexico; maricarmen.im@tec.mx (M.I.-M.); reyna.g@tec.mx (R.B.G.-G.); eldafc@tec.mx (E.A.F.-C.); rafael.araujo@tec.mx (R.G.A.); hafiz.iqbal@tec.mx (H.M.N.I.); r.parra@tec.mx (R.P.-S.); 2 Institute of Advanced Materials for Sustainable Manufacturing, Tecnologico de Monterrey, Monterrey 64849, Mexico; 3 Food Science and Technology Programme, Nanyang Technological University, 62 Nanyang Drive, Singapore 637459, Singapore; wnchen@ntu.edu.sg; 4 School of Chemical and Biomedical Engineering, Nanyang Technological University, 62 Nanyang Drive, Singapore 637459, Singapore; 5 Institute of Advanced Materials for Sustainable Manufacturing, Tecnologico de Monterrey, Tlalpan, Mexico City 14380, Mexico; mariel.alfaro@tec.mx

**Keywords:** edible coatings, essential oils, postharvest preservation, emerging technologies, berries

## Abstract

Berries are highly perishable and susceptible to spoilage, resulting in significant food and economic losses. The use of chemicals in traditional postharvest protection techniques can harm both human health and the environment. Consequently, there is an increasing interest in creating environmentally friendly solutions for postharvest protection. This article discusses various approaches, including the use of “green” chemical compounds such as ozone and peracetic acid, biocontrol agents, physical treatments, and modern technologies such as the use of nanostructures and molecular tools. The potential of these alternatives is evaluated in terms of their effect on microbial growth, nutritional value, and physicochemical and sensorial properties of the berries. Moreover, the development of nanotechnology, molecular biology, and artificial intelligence offers a wide range of opportunities to develop formulations using nanostructures, improving the functionality of the coatings by enhancing their physicochemical and antimicrobial properties and providing protection to bioactive compounds. Some challenges remain for their implementation into the food industry such as scale-up and regulatory policies. However, the use of sustainable postharvest protection methods can help to reduce the negative impacts of chemical treatments and improve the availability of safe and quality berries.

## 1. Introduction

The world’s population is nearly 8 billion people, with a projected increase to 9.8 billion in 2050; linked to this, the per capita consumption also is increasing, and the demand for fresh, minimally processed, and high-value foods is rising in tandem [1,2]. The intake of vegetables and fruit is important to have a human healthy lifestyle. High-nutritional foods require greater resources to address their production and handling until they reach the final consumer [3]. As a result, they have environmental impacts, such as deforestation and water and soil contamination, that should be addressed [4]. Agriculture productivity is significantly hampered by the continuing degradation of soils brought on by the increasing output. Since 2020, the percentage of people who experience hunger has increased from 9.2 to 10.4%, putting further strain on the agriculture and food security sectors [2,5].

The availability of fruits is vast; however, the berries group has particular importance due to their contents of phytochemical compounds (flavonoids, tannins, and phenolic acids) linked with biological activities such as anti-inflammatory, anticancer, antimicrobial, neuroprotective, and antimutagenic [6,7,8,9]. This is supported by epidemiological studies that report a protective effect of berry consumption against chronic diseases [10,11]. Berries are a diverse group of small size, sharp color (red, blue, or purple), soft texture and characteristic flavor, and highly perishable fruits that are cartilaginous endocarps full of seeds [12]. Commercial berries include strawberries, currants, gooseberries, blackberries, raspberries, blueberries, cranberries, grapes, and others less well-known such as boysenberries, bilberries, Jost berries, cloudberries, loganberries, and lingonberries. The berries’ structures differ depending on whether they are formed from a single or several fused fertilized ovaries, being categorized as simple (e.g., blueberries, cranberries) and aggregate (e.g., strawberries, raspberries, blackberries) fruits, respectively [12,13]. The major producers of berries are China, the United States (US), Mexico, Poland, and Germany. The global production of berries in 2021 reached 89.10 million tons [14].

Fresh, frozen, or processed berries, such as those used to make jams, juices, purees, syrups, and wines, are all consumed. However, other procedures, such as the thermal and irradiation techniques used to produce fresh and processed berries, respectively, might cause nutritional losses. [11]. Additionally, fresh berries are quite perishable and highly susceptible to suffering contamination by pathogens and spoilage microorganisms, generating great economic losses and health problems such as food poisoning [15]. Over time, several physical and chemical techniques, such as the use of pesticides, have been employed to mitigate these losses [2,13]. Moreover, the high-value market of these fruits promotes the constant search by scientists and the industrial sector for green alternatives to prevent deterioration and extend the postharvest shelf life of berries, aiming to achieve the worldwide distribution of premium-quality berries. In line with this, nanotechnology and artificial intelligence (AI) have significant roles in the preservation of berries. Nanotechnology can be used to enhance food packaging, creating a protective layer that slows down the spoilage process and reduces moisture loss [16]. This is achieved by the incorporation of nanoparticles adding antioxidants and antimicrobial compounds into the packaging material; which helps to maintain the berries’ quality and nutritional value [17,18]. Otherwise, AI algorithms can analyze the data from the environmental conditions used during the preservation process, such as temperature and humidity levels, to optimize the packaging used in the preservation procedure or predict the shelf life of the berries. AI also helps to automate the process, aiming to reduce the risk of human error while increasing the efficiency of the preservation processes [19,20]. The use of these technologies for berry preservation is an alternative to reduce waste and improve food safety concerns regarding these fruits. This article is focused on describing the traditional methods such as the use of “green” chemical, physical, and microbiological alternatives for the postharvest preservation of berries. Otherwise, emergent technologies are being introduced and valorized for fruit application, for example, nanotechnology, pulsed electric fields, and AI. The trends and challenges in the implementation of these preservation techniques at a large scale to achieve sustainable production aimed at a circular economy are reviewed.

## 2. Main Factors Causing Postharvest Losses of Berries

**Figure 1:**
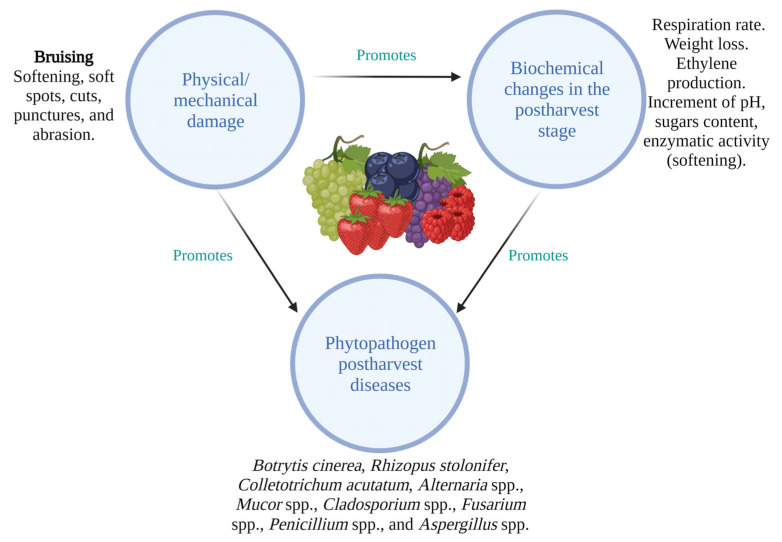
Main factors associated with the postharvest loss of berries.

Global fruit losses are estimated between 25 and 50% of total production [21]. Even under ideal storage conditions (−0.5 to 0 °C, 90–95% relative humidity, RH), blackberries and raspberries, for example, have a shelf life that can last only 2 to 5 days [13]. Berries are more susceptible to decomposing because of physical (mechanical damage, storage temperature), physiological (high respiration rate (52–245 mg CO_2_/kg h at 20 °C), fragile, ethylene production), and microbiological factors during harvesting, packaging, and transportation (Figure 1). Berries also have a high water content and water activity [12,22].

### 2.1. Physical Factors 

The perishable nature of berries during the postharvest stage is related to their susceptibility to physical damage during the whole supply chain, due to their softness and the lack of a protective layer [23]. The type of berry, fruit maturing, harvest time (during the day or season), amount of time following harvest, and storage conditions (temperature, humidity, etc.) are only a few of the variables that might cause physical damage to berries [2,8,24]. The most common physical damage to berries is named bruising, which is a type of physical damage that occurs when fruits and vegetables are subjected to external forces or pressure. This pressure can cause injury to the cells and tissues of the fruit, leading to discoloration, soft spots, and reduced shelf life. Bruising can occur at any production stage, from harvesting and handling to transportation and storage and can affect both the appearance and quality of berries [12,23]. This event causes discoloration in berries, leading to a loss of aesthetic appeal and marketability. Other mechanical damages that cause significant economic losses due to downgrading or rejection of the appearance quality by the consumer include cuts, punctures, splits, and abrasions in berries [2,12,23].

Berries are prone to physical damage caused by temperature in diverse ways. Freezing damage occurs when berries are exposed to temperatures below freezing; the water inside the cells can freeze and expand, causing cells to burst. As a result, berries become mushy and lose their texture and flavor. Conversely, heat-damaged berries have been exposed to high temperatures (>35 °C), and their cells can become damaged and break down, resulting in soft, discolored, and less flavorful berries [13,25]. Whereas, if berries are exposed to direct sunlight for extended periods, they can become sunburned, causing the loss of sensory characteristics such as color and flavor. Overall, it is important to handle and store berries carefully to avoid physical damage from temperature changes. This includes keeping them in a cool, dry place, avoiding exposure to direct sunlight, and minimizing temperature fluctuations [12,13,23].

To minimize physical damage to berries, it is important to use appropriate harvesting, handling, transport, and storage techniques. This may include using proper tools and equipment, gentle handling of the fruit, appropriate packing materials and containers, suitable loading and unloading techniques, and regular inspection and removal of damaged fruit. Additionally, maintaining optimal temperature and humidity levels and providing ventilation during storage can also help to minimize physical damage and prolong their shelf life. In general, minimizing physical berry damage is crucial for preserving fruit quality, increasing shelf life, and decreasing food waste throughout the supply chain [13,25]. It is crucial to remember that any physical damage encourages the growth of spoilage bacteria in the subsequent supply chain processes. Furthermore, physical damage could promote physiological changes leading to the rejection of the berry fruits by the consumers [12,23].

### 2.2. Biochemical Changes in Berries 

During the postharvest stage, berries undergo a series of biochemical changes that can significantly affect their quality, shelf life, and nutritional value. These changes are influenced by various factors such as genetic background, cultural practices, harvesting time, and storage conditions during postharvest handling such as temperature and relative humidity [26,27]. Respiration, a fundamental metabolic process, results in the utilization of sugars, which can alter the sweetness and flavor of berries owing to the formation of organic acids [28]. Anthocyanins, which are responsible for the bright red, blue, and purple hues of the berries, can be broken down due to pH change, resulting in a loss of color intensity [22,26]. In addition, the enzymes present in the berries become more active, leading to the breakdown of pigments and polymers [29]. Degradation of the cell walls may occur, resulting in softening of the berries and a change in texture. This is mainly driven by enzymatic processes carried out by the enzymes pectate lyases (PL), pectin methylesterases, and polygalacturonase that break down cell wall components such as pectin and hemicelluloses [30,31]. Most fleshy fruits may soften as they ripen, which is a common sign of fruit ripening. However, excessive softening harms fruit quality and causes it to lose its sensory and nutritional qualities, leading to the consumers’ rejection. These biochemical changes must be carefully managed in the postharvest phase to ensure that the berries retain their desirable characteristics and their shelf life is extended.

### 2.3. Microbiological Agents 

As berries reach full maturity, they become a great medium with high moisture levels containing simple sugars and essential nutrients for the development of spoilage microorganisms. Due to their high water content, nutritional profile, and low pH, berries deteriorate similarly to the majority of fruits and vegetables [32]. The main berry phytopathogenic fungus is the grey mold *Botrytis cinerea*, followed by *Rhizopus stolonifer*, *Colletotrichum acutatum*, *Alternaria* spp., *Mucor* spp., *Aspergillus* spp., *Penicillium* spp., *Fusarium* spp., and *Cladosporium* spp. [33]. *B. cinerea* attacks berry fruits at temperatures ranging from 15 to 25 °C and 95% RH. Infected fruits exhibit signs such as softening, dehydration, and the formation of grey mycelium [34]. Symptoms of anthracnose caused by *Colletotrichum* spp. are visible with the ripening of the fruit and cause pink-colored lesions, culminating in fruit senescence. Whereas *Rhizopus* spp. synthesizes pectic enzymes, which promote cell wall disruption due to pectin degradation, releasing water and, at the final stages, causing the formation of a white mycelium. Instead, *Alternaria* spp. can enter the epicarp through lesions or holes that occur naturally in the fruit. The development of black lesions with white or greenish gray mycelium indicating deterioration characterizes the disease caused by this fungus. *Alternaria* spp. grows between 20 to 30 °C and produces mycotoxins such as alternariol [33,35].

Other fungi that cause diseases in berries during the postharvest stage include *Phytophthora cactorum*, *Phytophthora capsica*, and *Lasiodiplodia theobromae* in strawberries, *Neofusicoccum algeriense* producing dieback in raspberries, and *Neofusicoccum ribis* and other species of this fungus in blueberries [33,36]. Postharvest diseases due to phytopathogenic fungi are the result of latent infections that are initiated during the preharvest stage with the dispersion of the conidia by the wind. Furthermore, injuries during harvest and the handling processes make them susceptible to infection by saprophytes microorganisms [37]. Usually, their control is carried out by synthetic fungicides, which harm human health and the environment; additionally, these compounds can promote the development of microbial resistance due to the constant exposition or the emergence of new biotypes [12,38]. For this, natural alternatives such as green chemical compounds, natural coatings, and biocontrol are being widely used [2,39,40]. The proper selection of the postharvest preservation method should be based on fruit requirements addressing the maintenance of two essential points: food safety (chemical, microbiological, and radiological contamination) and the preservation of quality parameters such as flavor, texture, appearance, taste, and nutritional value [8,41]. Ideally, a clear comprehension of the contributing causes of postharvest losses will help to reduce the damage. Additionally, the physiological state of the berries at harvesting time also affects their shelf life [2,39]. The traditional and recent technologies used for the postharvest protection of berries are reviewed in the next section.

## 3. Brief View of Traditional Methods of Microbial Growth Control: Fungicides

Fungicides are chemical substances used to control fungal diseases. Azoxystrobin and pyrimethanil are two of the most widely used fungicides in berries against *B. cinerea*, *Alternaria tenuissima*, and *Colletotrichum* spp. [33]. Azoxystrobin binds to cytochrome B, inhibiting electron transport between cytochromes B and C, suppressing mitochondrial respiration [42]. Instead, pyrimethanil inhibits methionine (an essential amino acid) biosynthesis by inhibiting cystathionine γ-synthase and cystathionine β-lyase. Furthermore, it inactivates extracellular enzymes such as cellulase and pectinase of *B. cinerea,* which produce fruit rotting [43]. Sulphur dioxide, a compound generally recognized as safe (GRAS), is employed as a gaseous disinfectant in berries to limit contamination by *B. cinerea* and prevent fruit browning by inhibiting enzyme reactions [12]. This substance is used in blueberries and grapes, and it is effective at concentrations ranging from 8 to 15%. Sulphur dioxide can be applied using tiny sachets inside the packing to delay fruit rotting by inhibiting enzyme-catalyzed reactions in spoilage microorganisms [44]. Even after remarkable results in fruit protection and quality, chemical treatments have serious consequences for the environment and human health [33]. For example, in neural cells, fungicides such as azoxystrobin quickly can constrain oxidative respiration and change the amount of lipids, producing neurotoxicity [45]. That is why there is an increase in the research into biological techniques for disease control [2].

## 4. Sustainable Alternatives for the Postharvest Protection of Berries

**Figure 2:**
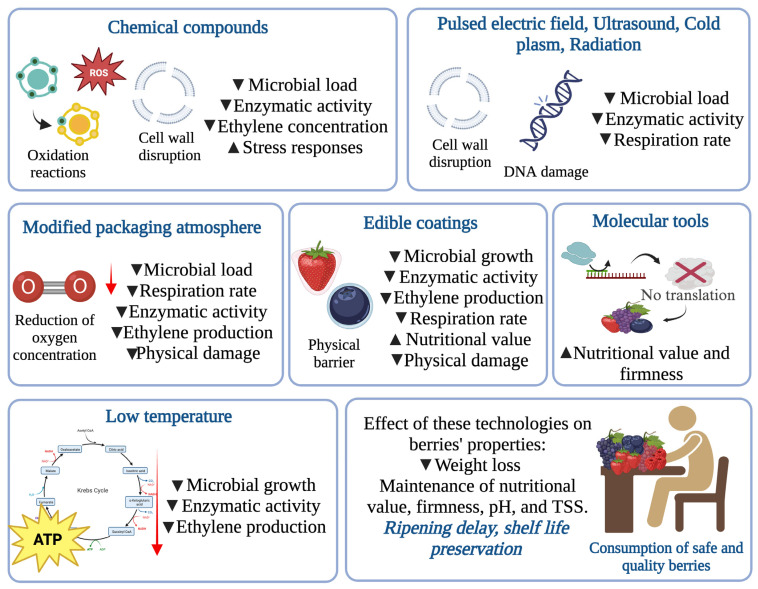
Action mechanisms and effects of different sustainable alternatives in berry preservation. The symbols ▲ and ▼ indicate increment and reduction, respectively.

The postharvest protection of berries is important to preserve their quality, extend their shelf life, and reduce losses due to spoilage and diseases. Sustainable alternatives for the postharvest protection of berries are important to reduce the negative environmental and health impacts of traditional methods. Alternatives to preserve berries and increase their shelf life include biological control agents, natural plant extracts, modified atmosphere packing, cold storage, ultraviolet (UV) radiation, and tools based on molecular biology (Figure 2) [12,46].

### 4.1. Green Chemical Compounds 

#### 4.1.1. Ozone

Ozone is a powerful oxidant that can be used as a gas to control postharvest diseases and maintain the quality of berries, particularly blackberries, blueberries, and raspberries [6,47]. The action mechanisms of ozone in the postharvest stage of berries include direct damage to fungal spores and bacteria cells by modification of the membrane permeability through the phospholipids oxidation [48]. Ozonolysis refers to the breakdown of alkenes bonds in polyunsaturated chains; then, these compounds are cleaved into organic radicals, peroxides, and aldehydes [49]. Ozone treatment can effectively control postharvest diseases such as grey mold and anthracnose (Table 1). Additionally, this compound delays fruit senescence and ripening by reducing the enzymatic activity and oxidating ethylene leading to a longer shelf life and the improved nutritional value and quality without compromising the flavor and texture of the fruit [6,13]. However, it is important to optimize the treatment conditions to minimize any potential negative effects, such as ozone-induced damage to the fruit surface [13]. Strawberries of the varieties Camino Real and San Andreas were treated with ozone (0.3 and 1 ppm) and stored at 10 °C for 12 days. The lower concentration showed a better effect on physicochemical (weight loss, firmness, color, pH, and total soluble solids—TSS) and microbiological (mesophilic aerobes, fungi, and yeasts) properties and improved total phenolic compound content in comparison with strawberries treated with 1 ppm. However, the concentration of 1 ppm hurt the physicochemical properties of both varieties, more markedly in San Andreas [46]. Hence, the response to a compound can vary between the different cultivars; for this reason, the appropriate validation of each treatment should be carried out aiming to reduce the economic losses while maintaining the quality parameters.

#### 4.1.2. Hydrogen Peroxide

Hydrogen peroxide (H_2_O_2_) can be used as a postharvest treatment for berries to protect them against decay and extend their shelf life. The action mechanism of H_2_O_2_ involves its ability to break down into reactive oxygen species (ROS) in the presence of enzymes such as catalase, peroxidase, and superoxide dismutase. ROS can oxidize lipids, proteins, and nucleic acids, leading to cellular damage and dysfunction [50]. H_2_O_2_ effects vary from minor oxidative stress to cell death depending on the concentration and exposure time of the H_2_O_2_. Moreover, at low concentrations, H_2_O_2_ acts as a signaling molecule, activating various pathways in the cell that regulate growth, development, and stress responses [51]. This means that the berries can protect themselves against environmental stressors and diseases that could lead to decay. In addition to this, H_2_O_2_ is shown to reduce ethylene production in berries, which helps to slow down the ripening process and extend the shelf life of the berries to maintain quality attributes such as color, texture, and flavor [50,51]. H_2_O_2_ was tested on fruits such as strawberries, red bell peppers, and watercress at higher concentrations (1 and 5%) for the assessment of the inhibition of *Listeria innocua*, coliforms, and mesophilic aerobes. H_2_O_2_ at 5% had a higher microbial reduction but produced color alterations, modified the sensorial properties, and decreased the anthocyanin content of strawberries [52]. There is not an established concentration for the use of H_2_O_2_ alone [53], but it is important to consider the effect of the high concentrations on the sensorial properties of the fruits.

#### 4.1.3. Peracetic Acid (PAA)

PAA is a powerful oxidizing agent used as a sanitizer for fresh produce, including berries, during postharvest handling. When used properly, it can effectively reduce the population of microorganisms on the surface of the berries and help extend their shelf life [41,54]. This reduction is achieved by the formation of radicals (^●^OH) that oxidate proteins, enzymes, and DNA. This process disrupts the structure and function of these components, eventually leading to cell death [55]. The action of PAA is concentration-dependent, and higher concentrations are more effective in killing microorganisms. The allowed concentration used by PAA to disinfect fruits and berries must not exceed 80 ppm in wash water [53]. However, like other disinfectants, it was tested in several ranges, which are slightly higher than the permissible limit. PAA at 24 and 85 ppm was tested to control *B. cinerea* and *Alternaria* spp. in blueberries stored between 0 and 1 °C for 4 weeks. As was expected, when using a higher concentration, the disease was reduced between 12 and 17% in comparison with untreated fruits without modifying their sensorial properties [41]. However, the use of PAA on berries must be carefully controlled, as excessive exposure or concentration can cause damage to the berries, including discoloration, texture changes, and even chemical burns [56]. In addition, the residue of peracetic acid on the berries may also affect the sensory characteristics of the fruit, including taste and aroma. In line with this, treatments with PAA at 80 ppm reduced the anthocyanins content in strawberries, whereas at 20 and 40 ppm, it did not affect any quality parameter. Additionally, after 2 min of exposure, all the assessed concentrations were reduced by more than 4 Log_10_ CFU/g of *Listeria innocua* [54]. Therefore, it is important to use peracetic acid according to the manufacturer’s instructions. Furthermore, more research related to the effect of PAA on the quality parameters of berries should be addressed. Because few studies have focused on the assessment of the effect of this compound on the sensorial properties, demonstrating that under the assessed conditions, the PAA does not modify the taste, firmness, and other sensorial properties of the fruit is essential [41].

**Table 1:** Postharvest preservation of berries by green alternatives.

Berry	Preservation Technique	Storage Conditions	Main Result	Reference
Blueberries	Peracetic acid (PAA, 85 µL/L)	1 °C/4 weeks	Inhibition of *Botrytis cinerea* maintaining the quality parameters of the fruit during the storage.	[41]
Blackberries and grapes	Ozone (18 mg O_3_/L for 10 min)	4 °C/ 20 days	Reduced fungal decay and loss of weight along with storage.	[47]
Strawberries, raspberries, and blueberries	Ozone (13 mg/m^3^ for 16 h at 1 ± 0.5 °C) and MAP (10 kPa O_2_ and 40 kPa CO_2_)	4 °C/15 days	The treatment did not affect the quality parameters of the fruits. In the case of blueberries, it protected the total and individual content of anthocyanins.	[6]
Strawberries	γ-irradiation (2 kGy, at 0.5 kGy/min)	4 °C/15 days	The antioxidant activity increased in comparison with untreated fruits.	[7]
Strawberries	γ-irradiation (2 kGy)	4 °C/14 days	Decreased the proliferation of molds and yeasts; sensory and physicochemical scores were not affected in comparison to the non-treated.	[8]
Goji berry	γ-irradiation (10 kGy, at 2.6 kGy/h)	5 °C/50 days	Irradiation increased antioxidant activity by almost 30% in comparison with untreated fruits.	[24]
Blueberries	Cold plasma (4 kV/10 min)	25 °C/10 days	Reduced decay, maintaining the quality and anthocyanin content of the fruits during storage.	[57]
Blueberries	Cold plasma (45 kV/50 s), ultraviolet (UV-C, 2.76 kJ/m^2^), or aqueous ozone (0.3 mg/L/5 min)	20 °C/8 days	Cold plasma was the most effective treatment in the maintenance of the quality parameters, inhibiting the fungal decay and the growth of the microflora.	[58]
Strawberries	Electron beam irradiation (2 kGy, 70 cm/min)	4 °C/14 days	Guaranteed microbial safety for up to 7 days and improved the physicochemical and sensorial properties of the coated fruits.	[59]
Strawberries, blackberries, and raspberries	Biodegradable packaging of gelatin- carboxymethylcellulose added with avocado peel extracts.	25–28 °C/6 days	Protected the fruit from fungal growth during storage.	[60]
Blueberries	Biodegradable packaging based on polyvinyl pyrrolidone and carboxymethyl cellulose added with bacterial cellulose and guar gum.	21 °C/15 days	Maintained the color and structure of the fruits after the storage period.	[61]
Blackberries and raspberries	Biodegradable packaging of poly (lactic acid) added with cyclodextrin and thymol.	4 °C/ 21 days	Prolonged shelf life by one more compared with commercial clamshell packaging, this means 21 days.	[62]

#### 4.1.4. Organic Acids

An organic acid is a compound that contains one or more carboxyl (-COOH) functional groups. Organic acids are commonly found in nature, in both plants and animals, and they play important roles in biological processes. Organic acids can be classified as either weak or strong, depending on their ability to donate hydrogen ions (H^+^) in aqueous solutions [56,63]. As a result of this event, organic acids reduce the environmental and cellular pH of microorganisms, which eventually causes the cell to die. Some examples of organic acids used to preserve the quality of berries include citric, ascorbic, malic, acetic, and lactic acid [56,64]. These weak acids are naturally found in fruits and vegetables (except lactic acid, which is a mild acid produced during the fermentation of dairy goods and vegetables) and are widely used to prevent spoilage and reduce browning while enhancing the flavor and aroma of berry fruits [64]. The application of organic compounds in berry preservation enhances the natural taste of berries and promotes color retention by preventing enzymatic browning, which occurs due to the oxidation of polyphenols in berries. Hence, the use of organic acids in berries could make them more appealing to consumers [11,64]. However, few studies were focused on the study effect of organic acids on berries; therefore, further research is needed to evaluate the safety and effectiveness of these chemical compounds on a larger scale and in different contexts.

### 4.2. Bioactive Compounds

Bioactive compounds such as essential oils (EOs) and plant extracts were extensively studied due to their outstanding properties, including their antimicrobial, antioxidant, and nutritional properties [65]. Those advantageous characteristics are usefully employed in the development of novel protective coatings for perishable food products. In this manner, different bioactive compounds were extracted for their evaluation and further application, to describe the effect of their composition, concentration, and extraction techniques, among many other influential parameters.

#### 4.2.1. Essential Oils (EOs)

EOs are effective and eco-friendly compounds frequently used for protection from postharvest diseases and to maintain the quality of berries. Several studies have demonstrated that their incorporation in coatings increases the storage time of fruits and protects them from microorganism growth caused by their antimicrobial and antioxidant properties [17,18,66]. Specifically, EOs can protect berries against weight loss, visible decay, and firmness loss, which are crucial quality parameters evaluated during the development of postharvest storage systems [67]. For instance, gelatin- and starch-based edible coatings enriched with cinnamon oil were evaluated for the maintenance of the selected quality parameters of blueberries. The authors observed multiple benefits, including a reduced loss of soluble solids and a greater inhibition of yeast and fungi, which, in turn, had a positive effect on the preservation of the fruit during storage time [4]. Some studies evaluate multiple quality parameters and the role played by EOs in performance enhancement. For example, weight and firmness loss are related to the vulnerability of perishable foods to fungal spoilage, which synthesizes enzymes that catalyze degradative reactions [65]. The prevention of weight loss is attributed to the inhibition of pathogenic fungi that produce pectinases, a type of enzyme that degrades pectic polysaccharides during the softening and ripening of fruits [2,68]. In this respect, strawberries exposed to carvacrol exhibited a higher firmness in comparison to untreated strawberries because the EO reduced the production of extracellular pectinase by *B. cinerea* [65].

The broad-spectrum fungitoxicity and antioxidant properties of different EOs, as well as the influence of some parameters (e.g., concentration) on the protection of berries have been demonstrated. Similarly, the effect of the droplet size of lemongrass oil (LO) was analyzed in terms of effectiveness in improving antimicrobial activity. Oh et al. [66] prepared emulsion coatings containing chitosan/LO nanoparticles of different sizes. Interestingly, they observed that the emulsion with the lower particle size (204–378 nm) presented enhanced results in comparison to the larger one (461–632 nm). The optimal emulsion showed higher antimicrobial activity against *Salmonella* Typhimurium; increased the growth inhibition of total mesophilic aerobes, fungi, and yeasts; as well as enhanced the results in terms of color retention, antioxidant activity, and sensory attributes [66]. Otherwise, the antioxidant capacity of berries can be enhanced by the addition of EOs. In this regard, blueberries coated with cinnamon oil exhibited a lower production of ROS, which was 82% lower than that obtained by the control. Accordingly, the activity of catalase and superoxide dismutase were lower than the control by 63 and 56%, respectively. In this manner, the antioxidant properties of berries can be maintained to minimize the damage caused by ROS. Therefore, the antioxidants contained in the EOs can slow down the loss of color, flavor, and nutritional content, thus, maintaining the overall quality of the berries [4]. However, it is important to assess the proper concentration of the EOs, aiming to avoid altering the sensorial properties of the foods [40].

#### 4.2.2. Plant Extracts

A plant extract is a concentrated solution of compounds derived from different parts of a plant, such as the stems, leaves, flowers, or roots. These compounds can be extracted using various methods, such as solvent extraction or steam distillation [69]. These extracts are used in a wide range of applications, including the pharmaceutical postharvest protection of fruits such as berries by dipping them in a solution of the extract or by spraying the extract on the surface [60,70]. Many plant extracts have been incorporated into protective coating systems to control postharvest diseases in berries. As a representative example, the effect of *Prosopis juliflora* water-soluble leaf ethanolic extract alone and combined with chitosan was assessed for extending the shelf life of strawberries. The optimal results were obtained when strawberries were coated with 8 mg/mL of the plant extract, either individually or in combination with 1% chitosan. The coating reduced weight loss and inhibited the growth of bacteria, yeast, and fungi while maintaining firmness and soluble solid levels. Hence, this plant extract provided beneficial effects for extending the shelf life of strawberries under room temperature conditions and even more at 4 °C [70]. On the other hand, avocado peel extract was used as a bioactive agent for the preservation of strawberries, raspberries, and blackberries. Avocado peel ethanolic extract contains bioactive compounds such as catechin hydrates, procyanidins, kaempferol, and epicatechin gallates, among others; providing antioxidant, antimicrobial, and antiradical properties [60]. The active packaging material exhibited great potential for the preservation of berries since the plant extract caused an increased antioxidant capacity and enhanced antifungal activity against *A. niger* and *R. stolonifer* [60].

Other plants have been used to obtain extracts for the same purpose; for example, lotus leaf was added to protective coatings for fresh goji fruit [71]. The flavonoids and phenolic compounds contained in the extract were reported to be responsible for the strong antioxidant and free radical scavenging capacity [71,72]. Similarly, extracts from *Aloe vera* have shown potential in the postharvest treatment of berries; after the addition of 300 mL/L of extract, the percentage of spoiled berries was significantly lower in comparison to that obtained in the control [73]. The antioxidant properties and antifungal capacity of *A. vera*, in addition to its nutritional properties, make it an excellent alternative for extracting bioactive compounds to protect fruits; therefore, *Aloe vera* was extensively studied, demonstrating its potential for extending the shelf life of different berry fruits [74,75]. The antioxidant content in plant extracts can help prevent oxidative damage, helping to maintain the color, flavor, and nutritional content of the berries over time. Additionally, some plant extracts can enhance the flavor of berries by adding complementary or unique taste profiles. This can make preserved berries more appealing to consumers [76].

The advantages of adding plant-based bioactive compounds were extensively demonstrated; however, the current existence of some drawbacks such as the complex composition and instability over time should be noted. The composition of EOs or plant-derived products is extremely variable and complex; thus, active coatings enriched with plant-derived products possessing standardized and constant characteristics are hard to obtain [65]. In this context, the addition of single bioactive compounds instead of complex plant-derived products might represent a better alternative, until we have a comprehensive understanding of their interaction. Additionally, it is important to mention that the selection of the extraction process also affects the composition of the EOs and extracts. Hence, the properties and activity of these compounds can vary depending on operational conditions and extraction techniques.

### 4.3. Physical Methods 

Physical methods play a key role in preserving the quality and safety of berries by reducing microbial contamination, preventing physical damage, and controlling ripening. These methods involve controlled atmosphere packaging, cooling, hot water treatment, or the use of edible coatings [12]. The use of physical methods in berry preservation can have several benefits, including (i) improving food safety by reducing microbial contamination and preventing the growth of pathogenic bacteria that can cause foodborne illness; (ii) extending shelf life by slowing down the ripening process, reducing physical damage, and preventing decay; (iii) improving nutritional and sensory quality by reducing oxidation, preserving texture, and maintaining color and flavor; and (iv) reducing the use of chemical preservatives that can be harmful to human health and the environment (Table 1) [12,56].

#### 4.3.1. Controlled Atmosphere (CA) and Modified Atmosphere Packaging (MAP)

CA and MAP are both postharvest preservation techniques that involve altering the composition of the storage environment around fruits and vegetables to extend their shelf life. CA is aimed to slow down the natural aging and deterioration processes of fruits and vegetables by controlling the levels of O_2_ and CO_2_ [77,78]. In berries, CO_2_ is used in concentrations ranging from 15 to 20%, whereas O_2_ is used around 80%. A high O_2_ atmosphere (80% O_2_ + 20% N_2_) and CO_2_ atmosphere (20% CO_2_ + 20% O_2_ + 60% N_2_) were compared to determine their effect on the properties of strawberries stored at 0 ± 0.5 °C for 10 days. The results showed both atmospheres maintained fruit firmness and reduced weight loss and decay rate. However, the high O_2_ atmosphere preserved polyphenol content and cell integrity owing to lowering the superoxide and hydrogen peroxide levels. A PCA analysis demonstrated that treatment with high O_2_ and CO_2_ atmospheres affected oxygen and carbon metabolism, respectively [79]. The use of CA (70 kPa O_2_ + 20 kPa CO_2_) also promotes the maintenance of vitamin C and proanthocyanidin in strawberries after 20 days of storage at 5 °C. Whereas, with the use of 90 kPa O_2_ + 10 kPa CO_2_, the anthocyanins content decreased gradually after 12 days of storage. However, flavonols, phenolic acids, and ellagitannins also experienced increases up to 130% at 5–12 d of storage in samples exposed to both atmospheres [78]. As in other preservation alternatives, different cultivars can have a different response to the same treatment. The effect of different concentrations of CO_2_ were assessed on five blueberry cultivars (Aurora, Brigitta, Duke, Jersey, and Liberty). In general, the response of fruit firmness, sugar, and acid content was similar in all the cultivars. However, the production of volatile compounds was affected in different ways depending on the cultivar, Liberty showed a lower production of fermentation-induced volatiles, and Aurora had the least flesh discoloration. The use of high CO_2_ is effective to control the decay by microbial activity; its effects on fruit flavor due to the modification of the volatile production and changes in the ratio of sugar/acid must be considered in optimizing storage atmospheres [77]. However, CA by high O_2_ or CO_2_ is a powerful tool to prolong the freshness and quality of berries due to controlling the decay rate; however, their application should be optimized to each cultivar, aiming to reduce their effect on the sensorial properties of the fruits [77,78].

MAP is a technique used to extend the shelf life of berries by altering the gas composition around the fruit. The primary goal of MAP for berries is to maintain a low oxygen and high carbon dioxide environment, which can slow down the respiration rate of the berries and extend their shelf life. In the case of berries, the main gases used in MAP are carbon dioxide (CO_2_), oxygen (O_2_), and nitrogen (N_2_) [6]. The specific composition of the gas mixture may vary depending on the type of berry, its maturity stage, and other factors. Usually, the package’s O_2_ concentration is reduced to around 2–5%, while the CO_2_ concentration is increased to 5–20%. The remaining gas in the package is usually N_2_ [80]. The combination of these gases is chosen to suit the specific type of berry being packaged. Some of the benefits of MAP for berry preservation include (i) reducing microbial growth by creating an environment with low oxygen and high carbon dioxide; (ii) decreasing the respiration rate of the berries, which can reduce the production of heat, water vapor, and carbon dioxide; and (iii) minimizing the physical damage during transportation and storage by providing a cushion of gas that can absorb shock and prevent bruising [80,81,82]. Additionally, this method involves the use of specialized packaging materials that allow for the exchange of gases, such as O_2_ and CO_2_. Some examples of MAP packaging used for berry preservation include clamshells with small ventilation holes that allow for the exchange of gases that are widely used for strawberries [28]. Whereas raspberries are highly sensitive to moisture and require packaging that can prevent the accumulation of water vapor; therefore, they are often packaged in high-barrier plastic trays with lids that are sealed tightly to prevent moisture buildup [28]. Ethylene scavenger sachets that can absorb the gas and slow down the ripening process are widely used for blueberry preservation, which is prone to over-ripeness due to the high production of the mentioned gas [6,13]. Strawberry, red raspberry, and blueberry fruits were exposed to gaseous ozone (13 mg/m^3^ for 16 h) and subsequently stored in microperforated polypropylene bags under 10 kPa O_2_ and 40 kPa CO_2_ at 4 °C for 15 days. The combination of these treatments preserves the microbiological and nutritional quality of the berries. However, the effect was different for each fruit; for strawberries, the total anthocyanins increased up to 0.2 g/kg and the counts of molds and yeasts were significantly reduced [6]. MAP can slow down the respiration rate, reduce physical damage, and prevent microbial growth, preserving the texture, flavor, and color of berries. Specific MAP packaging can be used for different types of berries, depending on their specific preservation needs. Even with all benefits of MAP, its application in the supply chain of fruits and vegetables could be limited at the industrial scale by moisture condensation, which can promote fungal growth and food decay [83] 

#### 4.3.2. Low Temperature

Temperature is one of the most crucial factors influencing the storage shelf life and quality of berries since it affects the rate of all metabolic processes that occur in these fruits. Low temperatures decrease the fungal growth rate while reducing respiration rate and water loss, delaying the ripening and senescence processes. Berries are resistant to chilling harm; hence, prolonging their shelf life by lowering the temperature is common. The ideal storage conditions for strawberries, raspberries, and blackberries are 0 °C and 90–95% RH [13]. Because of the well-established favorable benefits of low temperatures on postharvest shelf life and the quality of berries such as TSS, vitamin C, and antioxidant compounds [4], storage at low temperatures in combination with other factors such as modified atmospheres is a powerful alternative for the postharvest protection of berries (Table 1). In agreement with this, the technology of multiple barriers was used for the preservation of strawberries, raspberries, and blueberries, combining storage at 1 ± 0.5 °C with using ozone (13 ± 1 mg ozone/m^3^) for 16 h and then at 4 °C under a MAP (10 and 40 kPa CO_2_) for 15 days. The established process preserved the microbiological and nutritional quality of the fruits during storage. However, the authors suggested that storage conditions cannot be generalized for the three fruits due to the variability in their chemical composition and requirements. Hence, particular conditions should be tested for each crop [6]. Cold storage is a common method used to extend the shelf life of berries. Low temperatures slow down the ripening process and prevent the growth of pathogens [4,13]. The selection of temperature storage should be carried out carefully, aiming to avoid the damages caused by improper storage. Care must be taken to avoid freezing, as this can damage the cell structure of berries and affect their quality.

#### 4.3.3. Ultraviolet (UV) Irradiation

UV irradiation is a non-thermal food processing technology that is used to preserve the quality and extend the shelf life of fresh fruits such as berries. UV irradiation affects the surface of the fruit, where most of the microorganisms are present. Its action mechanism is based on its ability to cause microbial DNA damage, disrupting its cellular processes, leading to its inactivation or death [84]. The UV-C wavelength range (200–280 nm) is the most effective for microbial inactivation. In addition to its antimicrobial effect, UV irradiation can also affect the physicochemical and nutritional properties of berries. It can stimulate the production of phytochemicals, such as anthocyanins and flavonoids, which are responsible for the color and antioxidant properties of berries [84,85]. However, excessive exposure to UV-C light can cause damage to the cellular components of the berries, reducing their quality and shelf life. To minimize the negative effects of UV-C irradiation, the treatment conditions, such as irradiation dose, exposure time, and distance from the source, should be carefully controlled and optimized [85]. All these responses were demonstrated using UV-C at 5.3, 8.3, and 11.4 kJ/m^2^ on O’Neal blueberries. The highest dose tested was the most successful in delaying infection for native microorganisms (six days) and *B. cinerea* (four days). The higher rupture force and deformation resistance values in irradiated fruits on the 15th day of storage at 8 °C were related to increased epicarp walls and detachment of the peel from the mesocarp, which could be due to a hormetic effect. The microstructural and ultrastructural characteristics indicated an effect of UV-C radiation on the waxes and cuticle, explaining the increment of approximately 2% of the weight loss observed in irradiated fruits in comparison with non-irradiated fruits. UV-C is an effective and reliable alternative to extend the postharvest shelf life of berries [86]. However, excessive dosage can cause damage and loss of quality properties in berries. Its use in combination with other methods may be considered.

#### 4.3.4. Pulsed Electric Field (PEF)

PEF is a technology that uses short pulses of high-voltage electric fields to improve food quality, safety, and shelf life. During PEF treatment, a strong electric field is applied to the food, causing the cell membrane to become permeable and allowing the exchange of ions and molecules between the inside and outside of the cell. This disruption of the integrity of the cell membrane results in the inactivation of microorganisms, enzymes, and other biological components in the food [87]. In berries, PEF treatment can have various effects on their quality, including (i) maintenance of nutritional quality through the reduction of the loss of nutrients during processing; additionally, this treatment can help break down the cell walls of the fruit, making nutrients more accessible and bioavailable (it is important to consider that this event accelerates the senescence stage); (ii) improved texture of berries by breaking down the cell walls and making them softer and juicier; and (iii) extended shelf life via inactivation of spoilage microorganisms and enzymes that can cause spoilage [87]. The combination of PEF (2 kV/cm electric field strength, 1 µs pulse width, and 100 pulses per second for 2 and 4 min) and PAA (60 ppm) was able to reduce *E. coli* and *Listeria monocytogenes* and *L. innocua* by up to 3 Log/g and native microbiota by 2 Log/g. The PEF treatments did not cause changes in the blueberries’ color and appearance. However, the treatments caused softening of the blueberries (119.1 ± 39.1 and 143.6 ± 44.2 g for treatments of 2 and 4 min, respectively) compared to the untreated fruit (499.8 ± 134.6 g). The anthocyanins and phenolic compounds in blueberries increased by 10 and 25%, respectively, after 4 min of PEF treatment [88]. PEF can help to reduce waste and improve the availability of fresh berries to consumers, but the conditions must be established for each berry type, aiming to reduce tissue damage.

#### 4.3.5. Cold Plasma (CP)

The action mechanism of CP on microorganisms is multifactorial, involving the generation of reactive species, such as O_3_, H_2_O_2_, and nitric oxide, which can cause damage to the cell membrane and intracellular components of microorganisms [89]. When CP is applied to postharvest stage berries, its antimicrobial and antioxidant properties reduce the microbial load on the surface of the berries. Additionally, it was also reported that it can affect the metabolism of fruits, preventing decay and extending their shelf life [19]. In line with this, CP was effectively used to maintain the quality and inhibit the growth of microorganisms in blueberries. Treatment preserved the firmness, sugar, and ascorbic acid of fruits, whereas metabolic assays revealed an increment in levels of chalcones, dihydroflavonols, dihydroflavones, flavanols, flavones, procyanidins, and anthocyanins but lower levels of L-phenylalanine than untreated fruits. The activities of key enzymes in the phenylpropanoid metabolism (phenylalanine ammonia lyase, chalcone synthase, and UDP-glucose:flavonoid 3-O-glucosyltransferase) were higher in the treated blueberries than in the controls. This suggests that CP affects the conversion of L-phenylalanine to phenols, flavonoids, and anthocyanins by regulating the mentioned enzymes [90]. CP also can produce softening in berries, making them more susceptible to damage [57]. However, this effect can be controlled by adjusting the treatment time and intensity of the cold plasma application [89]. The application of CP has the potential to improve the postharvest quality and shelf life of berries, but further research is needed to optimize the treatment parameters and to ensure the safety of the technology for human consumption.

#### 4.3.6. Ionized Irradiation 

Ionizing irradiation is a technology used to extend the shelf life of fresh produce, including postharvest berries. The action mechanism of ionizing irradiation involves the use of high-energy radiation, such as gamma rays, X-rays, or electron beams to create ionizing particles that can penetrate the tissues of the produce. The ionizing particles generated by the ionizing radiation have enough energy to knock electrons out of atoms, creating ions and free radicals and ROS in the production [7,13]. The radiation damages the DNA of microorganisms, rendering them unable to reproduce and, thus, reducing their numbers. As these molecules cause damage to microorganisms, they can also cause cellular damage, and disrupt normal physiological processes leading to changes in the texture, color, and flavor of the berries [7,8]. The severity of the damage caused by ionizing irradiation depends on several factors, including the type of radiation, the dose, and the duration of exposure. Exposure to ionizing radiation can induce a stress response in the berries, which triggers the production of antioxidants and other protective compounds, extending their shelf life. However, a decrement in citric acid content in irradiated berries was also reported [7]. Irradiations of 0.5, 1, and 2.5 kGy were tested to investigate their effect on intracellular Ca^2+^ concentration (which is related to firmness) in blueberries stored at 5.5 °C for 30 days. During the chilling period, intracellular Ca distribution had no effect on the fruit firmness of blueberries irradiated with 0.5 and 1 kGy. The subcellular Ca redistribution induced by irradiation with 2.5 kGy could promote the migration of intracellular Ca into the cell wall, which had a positive effect on the blueberries’ firmness [91]. Despite the potential benefits of ionizing irradiation for postharvest berries, there are also concerns about its safety and potential health risks. The World Health Organization (WHO) and Food and Agriculture Organization of the United Nations (FAO) established guidelines and maximum allowable doses (10 kGy) for food radiation [92], and these regulations must be followed to ensure the safety of the products and the consumers who eat them. The use of ionizing radiation should be accompanied by appropriate handling and storage practices to ensure the food’s safety and quality.

#### 4.3.7. Ultrasound (US)

The US is a non-invasive technology that utilizes high-frequency sound waves to generate mechanical energy that can be utilized for various applications in the food industry, including postharvest treatments of fruits and vegetables [93]. The action mechanism of the US is based on the principle of cavitation, which implies the formation and collapse of small bubbles or cavities in a liquid medium. When US waves pass through a liquid, they cause the formation of small gas-filled cavities or bubbles [93]. As the intensity increases, the bubbles grow larger and eventually collapse violently, generating localized high temperatures and pressures that can cause physical and chemical changes in the surrounding medium [94,95]. With this effect, the US was assessed for microbial inactivation in fresh products, such as blueberries, which demonstrated that the application of low (20 kHz) and high (1 MHz) frequency US for 10 min reduced ~2.75 Log_10_ CFU/g of *L. innocua*. These results suggested that the US, even at high frequency, provides limited control of pathogenic bacteria on the surface of the fruits. Additionally, as the value parameters increase, the probability of causing damage to berry tissue also increases, significantly reducing the firmness of blueberries after the treatment. Hence, the US should be combined with other treatments such as the application of carvacrol (2 mM) and carbonated water to increase the microbial reduction, which showed an increment of 0.5 Log_10_ CFU/g of *L. innocua* [93]. Additionally, in the case of postharvest berries, the US can improve their quality by affecting various physiological and biochemical processes. For example, it can enhance the activity of certain enzymes, such as pectin methylesterase, which can help to soften the berries’ tissue and improve its texture. It also increases the permeability of the cell membrane, which can facilitate the diffusion of various nutrients and bioactive compounds into the berries tissue, leading to increased nutritional and antioxidant content [95]. Hence, the US can extend shelf life and maintain the strawberries’ quality. However, its effectiveness on postharvest berries is influenced by several factors, such as the type and intensity of ultrasound, the duration of treatment, the temperature and pH of the treatment medium, and the stage of berry maturity [93]. Therefore, it is important to optimize these parameters to achieve the desired effects and to ensure that the treatment does not cause any negative effects on the quality and safety of the berries.

#### 4.3.8. Edible Coatings

Coatings differ from films in the fact that they are developed directly on the surface of the coated food, mainly by dipping or spraying [2]. Coatings contribute to extending the shelf life of berries by reducing the respiration rate, water loss, and gas exchange [2,96]. Edible coatings made of polymers such as polysaccharides, proteins, and lipids derived from animals or plants are important alternatives that help to offset the detrimental impact of synthetic films and coatings on the environment and human health [2,97]. Due to the abundance of biopolymers in renewable sources and their low price, a sizable number of studies have concentrated on the creation of biodegradable food packaging made of these materials. The most widely used biopolymers in the formulation of edible coatings for berries include chitosan, sodium alginate, cellulose, and pectin (Table 2). Biopolymers provide appropriate coating for each type of perishable food while retaining their sensory and nutritional qualities [9,98]. Additionally, these types of edible coatings can be functionalized to deliver various active ingredients to work and improve the stability, quality, and safety of coated perishable foods, such as antimicrobials, antioxidants, antioxidant agents, volatile precursors, nutrients, flavoring compounds, and coloring compounds [96,98]. 

Agro-industrial leftovers can be used to create some of these active compounds, which helps ensure the long-term survival of the sector. Additionally, the mechanical, structural, and water vapor and gas barrier properties of edible films and coatings are enhanced by combining two or more biopolymers to create complex coatings, as well as by adding plasticizers and/or cross-linking agents [16,100]. Additionally, the polymeric matrix can improve the activity of the biocontrol agents (BCAs) and bioactive compounds, improving their distribution on the surface of the coated fruit, protecting them against environmental conditions, promoting their adherence, and favoring releasing control. Finally, depending on the coating formulation, this can provide added nutritional value to the coated food. Chitosan coatings added with prebiotics (orange fiber, apple fiber, oligofructose, and inulin) extend the postharvest life of blueberries stored at 5 °C six days more in comparison with uncoated fruits. Controlling the microbial growth enhanced the antioxidant properties and maintained the quality parameters of the fruits, being an advantageous prebiotic product [106]. Each technology provides advantages in the extension of the shelf life of berries but also shows drawbacks that need to be considered during the selection of the preservation method (Table 3). Finally, it is important to mention that the use of edible coatings containing compounds from animal sources disqualifies the use of the term “vegetarian”, even in fruit products. Furthermore, it is crucial to bring attention to the formulation of the edible coating to berries obtained under an organic scheme [107].

### 4.4. Biocontrol Agents (BCAs)

Biocontrol is an innovative alternative that has been widely used in recent years, consisting of the use of bacteria, yeasts, endophytic fungi, or their products to prevent, reduce, or eliminate the development of pathogenic and spoilage microorganisms, their applications being possible via coatings or sprays [2]. BCAs inhibit the growth of other organism competition for space and nutrients, biofilm development, and the production of secondary metabolites, such as volatile organic compounds (VOCs), lytic enzymes, peptides, antibiotics, and the activation of plant defenses [2,109]. The first action mechanism used for BCAs such as bacteria and yeast is competition for nutrients and space, which have a time generation ranging from 0.3 to 2 h for being able to use the carbon sources efficiently for their survival and multiplication, limiting the availability of essential nutrients for the growth of phytopathogenic fungi [110,111]. The production of antimicrobial compounds such as active peptides, antibiotics, hydrolytic enzymes, and VOCs is the second most important mechanism used by BCAs [2,111]. The production, assessment, and alternatives to apply VOCs are being widely studied; however, most of the studies do not report the application of these compounds on fruits, even when it is reported that VOCs such as 2,4-di-*tert*-butylphenol produced by *Bacillus siamensis* G-3 remain in ~10% of the diseases caused by *B. cinerea* and *R. stolonifer* in raspberries stored at 0 °C for 20 days [112]. However, it is well known that the presence of multiple action mechanisms in BCAs improves the probability of reaching a higher control of spoilage microorganisms; for this, biocontrol is considered to be a dynamic process affected by the interaction of antagonist–pathogen–fruit [113]. Contributing to the effectiveness of BCAs tends to be lower in vivo than in vitro conditions; however, most of the studies are focused only on the in vitro assessment without the performance of the in vivo test. In line with this, *Bacillus subtilis*, *Bacillus licheniformis*, and *Leifsonia aquatica* inhibit up to 40% of the soft rot caused by *R. stolonifer* in blackberries. This was possible due to the convergence of several action mechanisms present in the tested bacteria. *Bacillus* species synthesize surfactants such as surfactin, initurin A, and amicoumacin, and additionally, both genera produce siderophores, VOCs, and enzymes [111]. Although the activity of BCAs can vary depending on the environmental conditions, biocontrol is a beneficial alternative to increase the shelf life of berries because it is effective in the short, medium, and long terms and does not harm the environment or the health of people or animals [109,111]. Additionally, BCAs could help to maintain the natural defense mechanisms of the berries during the postharvest stage without leaving residues, as occurs with chemical compounds [2,114,115]. It is important to mention that BCAs provide better preservation of fruits when applied in the postharvest stage because of their sensitivity to environmental conditions such as ultraviolet light, water limitation, nutrient limitation, temperature variations, and so on [2]. To improve their stability at the preharvest stage and contribute to the replacement of chemical compounds protecting BCAs, alternatives such as spray drying can be explored.

### 4.5. Molecular Tools to Improve Berry Preservation

Biotechnological tools encompass a wide range of techniques and technologies that leverage biological systems or living organisms to develop innovative solutions and products based on genetic modification. These tools have applications in various fields, including agriculture and food preservation [31,116]. The ripening and softening of fruits are two key factors in their perishability. In these processes, numerous biochemical process-regulated by well-coordinated genes are involved; regulating the expression of these genes is an opportunity to extend the shelf life of the fruits [30,117]. In line with this, antisense technology is a molecular tool that involves the use of synthetic oligonucleotides that are complementary to a specific mRNA sequence to selectively inhibit or downregulate the expression of a target protein. For example, the inhibition of PL genes for preserving fruit quality using antisense technology was assessed. Transgenic strawberry plants were obtained with an antisense pectate lyase gene under the control of a 35S promoter to control fruit softening. Forty-one transgenic lines were identified, of which six were selected for their transformation with the *pGUSINT* plasmid. The produced fruits with the transformed lines were firmer than non-modified strawberries, owing to the gene expression of the six PL lines being reduced by 30%, and three of them were suppressed in three lines. Hence, the use of antisense technology to reduce the expression of PL genes emerges as a prime candidate for enhancing strawberry softening through biomolecular tools [31]. On the other hand, pectin methylesterase, which catalyzes the pectin de-esterification, is regulated by RNAi-silencing of the *FvPME38* and *FvPME39* genes. As a result, the firmness of the assessed fruits was improved in comparison with the control [116]. Instead, the edition of *FaPG1* gen involved in polygalacturonase synthesis in strawberry plants cultivar Chandler was knockout using the CRISPR/Cas9 system delivered via *Agrobacterium tumefaciens*. Physical analyses showed that seven of the eight lines analyzed produced firmer fruits (33 to 70%) than the control. Additionally, modified fruits showed less transpiration water loss and were less susceptible to the disease caused by *Botrytis cinerea*. Finally, minor changes were observed in color, soluble solids, titratable acidity, or anthocyanin content [30]. The use of molecular biology tools is a promising approach to extend the shelf life and improve the quality properties of fruits. However, their implementation should consider factors such as safety, regulatory compliance, consumer preferences, and environmental impact.

## 5. Role of Artificial Intelligence (AI) in the Postharvest Protection of Berries 

One of the primary applications of AI in berry preservation is in the monitoring of environmental conditions. AI algorithms can be used to analyze data from sensors that measure temperature, humidity, and other factors that affect berry quality. By monitoring these conditions in real time, AI systems can identify any deviations from the ideal conditions and take corrective actions. For example, if the temperature rises above a certain threshold, the AI system could adjust the cooling system to bring the temperature back down [118]. The prediction of berry quality can be achieved with the use of AI by analyzing data on factors such as berry size, color, and sugar content; it is possible to estimate how long the berries will remain fresh and identify any potential quality issues [20,119]. The use of mathematical models based on image analyses and electronic devices coupled with instrumental equipment provides new opportunities to apply AI in fruits and vegetable preservation. Image-processing algorithms recently were examined for estimating the TSS and pH of strawberries. Multiple linear regression and support vector machine regression (SVM-R) models were developed using RGB, HSV, and HSL color-space channels as input variables. The findings showed that an SVM-R model trained on HSV color-space features outperformed an MLR model for TSS solids and pH prediction with an accuracy of 79.2 and 72.6% for TSS and pH at the testing stage, respectively [20]. On the other hand, the use of compounds derived from metabolism such as VOCs, which have a strong influence on the odor and taste of fruits and are critical sensory characteristics for consumer acceptance, also were tapped in this regard. The E-nose is an electronic device that simulates the human olfactory system, proving a digital VOC fingerprinting that can be processed by statistical tools. E-nose, in combination with attenuated total reflection-Fourier transform infrared spectroscopy and image analysis, was used as a fast and non-destructive methodology to distinguish between two ripening stages (half-red or red) of the strawberry cultivar “Sabrosa”, harvested at three different times. The principal components analysis performed revealed an association between the E-nose signals and the fruit maturity degree, which was confirmed by the physicochemical parameters. Demonstrating the sensitivity of an E-nose converts to a useful non-destructive technique to estimate the maturity stage of berries [119].

AI helps distributors make better decisions about transporting berries, reducing waste, and improving profitability. However, one of the primary challenges is the need for high-quality data. AI algorithms rely on large amounts of data to learn and make accurate predictions. Therefore, it is important to ensure that the data collected from sensors and other sources are accurate and representative of the conditions in which the berries are being stored. In addition to this, developing and implementing AI systems can be time-consuming and costly and requires expertise in data science and computer programming. Furthermore, there may be regulatory and ethical considerations associated with the use of AI in food production and preservation [19,25]. However, AI is the most powerful tool for improving berry preservation by providing more precise and efficient methods for monitoring and controlling environmental conditions.

## 6. Nanotechnology Applied to Postharvest Protection of Berries

**Figure 3:**
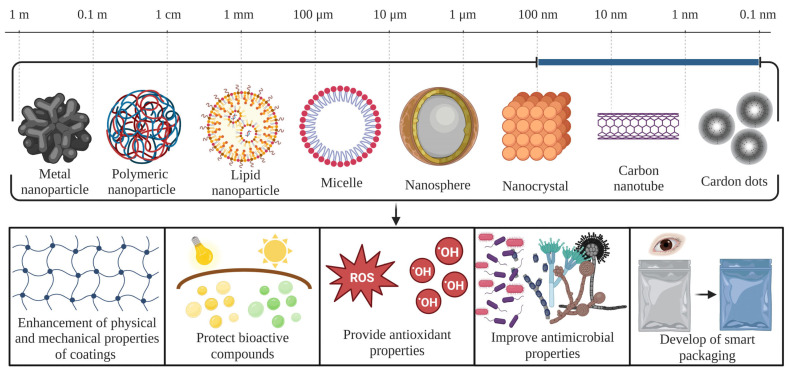
Main nanostructures used for berry preservation and their effect on coatings. The blue line indicates the range of the size of nanostructures.

Nanotechnology has great potential in the postharvest protection of berries, which is an area of increasing concern due to substantial losses and deterioration in the quality of fruits during the handling and storage process [17,18]. Researchers have applied nanotechnology to the postharvest protection of berries in various innovative ways to extend berry shelf life (Figure 3). The coatings made or added with nanoparticles from natural sources, such as chitosan or cellulose nanocrystals, provide a protective barrier against moisture loss, gas exchange, and external pathogens, thus, improving the fruit’s quality and extending its shelf life [17,103,120]. Nanomaterials made of chitosan ethyl cellulose, alginate, poly-ε-caprolactone, polylactic acid, poly-D, L-lactide-co-glycolide, and cellulose acetate phthalate, were used as antimicrobial agents to inhibit the growth of pathogenic microorganisms, including fungi, yeast, bacteria, and viruses, or to develop composite coatings to improve the shelf life of berries (Table 3) [17,103,120]. Furthermore, they provide multiple advantages to food coatings, such as the enhancement of mechanical properties and selectivity to gas permeability. The size of nanoparticles ranges from 10 to 1000 nm, and they are divided into two types, nanospheres and nanocapsules. The difference between each one depends on the way of carrying the target molecule. The nanospheres absorb the target molecule on its surface or it is dispersed in the matrix, while in nanocapsules, the target molecule is in the core [121]. These nanoparticles are usually loaded with ε-polylysine, curcumin, and phenols that have antimicrobial activity against *E. coli*, *S. aureus*, *Pseudomonas aeruginosa*, *Candida albicans*, *Bacillus cereus*, and *L. monocytogenes*. The action mode of these compounds to exert antimicrobial activity consists mainly of destabilizing the membrane and promoting the loss of intracellular content [122]. Moreover, nanotechnology-based edible coatings have been successfully used for the preservation of berries by the nanoencapsulation of EOs [4,17,18].

Another type of nanoparticle made from polymers is micelles, they usually have a size range of 400 to 800 nm. Micelles are made up of a hydrophobic nucleus that can transport antimicrobial compounds such as silver nanoparticles and EOs. Unlike micelles, lipid nanoparticles can carry hydrophilic (antimicrobial peptides) and hydrophobic (EOs) bioactive components in their core [123]. Lipid nanoparticles use solid lipids to have a solid and spherical structure with sizes ranging from 50 to 1000 nm [122]. Otherwise, metallic nanoparticles used for berry preservation usually are made of gold, silver, copper oxide, zinc oxide, and titanium oxide [9]. They cover sizes from 10 to 100 nm and are involved in improving the mechanical properties of coatings controlling water vapor permeability, promoting freshness of food, and increasing shelf life. In addition, these nanoparticles do not have a specific antimicrobial mechanism of action, avoiding the development of the resistance of microorganisms and causing the death of microorganisms such as Gram-negative/positive bacteria and fungi [18,100]. Nanocrystals, usually made of cellulose, are added to improve the mechanical and barrier properties of coatings. These rigid and narrow structures have antimicrobial activity against *S. aureus*, *E. coli*, and *P. aeruginosa* [124]. Their antimicrobial mechanism of action is unknown, but it is speculated that they perforate the cell membrane, causing the release of their intracellular content. In addition to this, nanocrystals are biodegradable, non-toxic, offer resistance to UV rays, are an excellent protective barrier against water and oil, and improve the antioxidant properties of food [18].

Currently, there has been growing interest in the application of carbon dots (CDs) in the preservation of berries and other fruits due to their unique properties. CDs are nanomaterials that have a size range of approximately 1–10 nm, and they possess excellent fluorescence properties, high stability, and biocompatibility [100,125,126]. These nanoparticles were shown to have a significant impact on the preservation of berries due to their ability to scavenge free radicals, which are produced during the process of fruit ripening and lead to a loss in fruit quality [100,125]. The antioxidant properties of CDs help to delay the ripening process, reducing the rate of spoilage, thus, improving the quality and shelf life of berries. Furthermore, CDs also have antimicrobial activity, which helps to inhibit the growth of pathogenic microorganisms that cause spoilage and foodborne illnesses, such as *Salmonella* and *E. coli* [125]. CDs properties can be successfully applied in the development of smart packaging, providing a real-time response on the quality properties of the packed fruits [126]. The application of CDs in the preservation of berries is a promising area of research with the potential to improve the quality and quantity of fruit production, reduce postharvest losses, and enhance food security. Other carbon-based nanomaterials are nanotubes, which have a cylindrical structure and are made up of rolled graphene sheets. Nanotubes are classified into two types: single-walled nanotubes (diameters from 1 to 3 nm) and multi-walled nanotubes (diameters ranging from 5 to 40 nm). These types of structures are usually used with matrices of polysaccharides and proteins and are mainly used to modify the mechanical properties (tensile strength and elasticity), provide thermal stability and improve the permeability barrier towards water vapor and oxygen in food packaging [122]. The use of nanotechnology in the postharvest protection of berries provides a sustainable alternative to conventional methods, essential for meeting the growing demand for high-quality fruits and vegetables, reducing postharvest losses, and improving food security.

## 7. Current State and Challenges in the Implementation of Sustainable Alternatives at the Industrial Scale for Berry Protection

**Figure 4:**
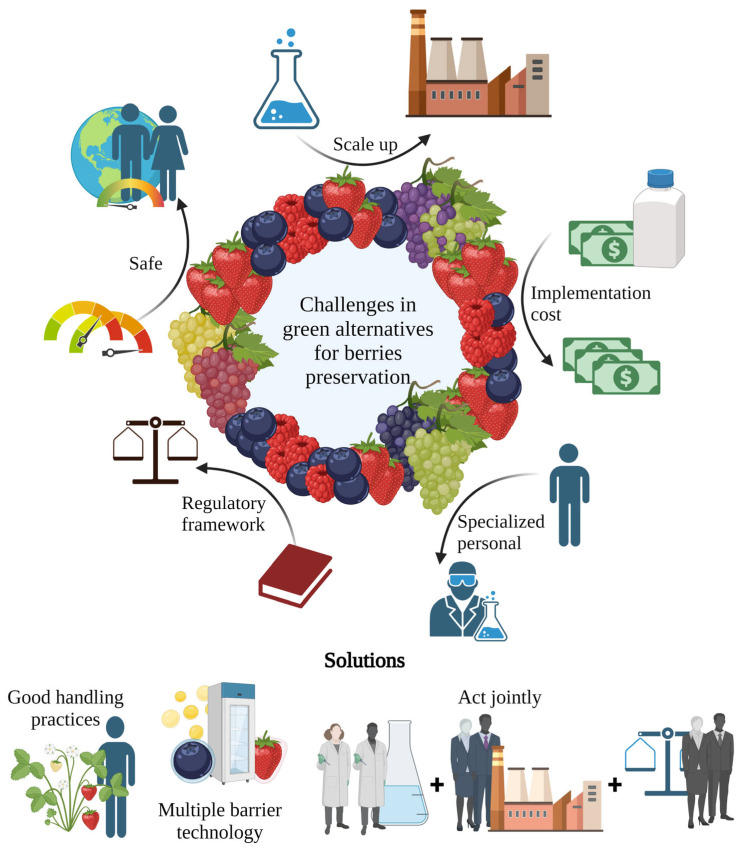
The main challenge for the implementation of sustainable alternatives for the postharvest protection of berries.

The rising concerns about synthetic fungicides and other chemical treatments’ negative environmental and health impacts have led to an increased interest in developing alternative solutions that are natural-based, such as the use of nanotechnology-based coatings and antioxidant compounds derived from plant extracts. There is a growing awareness of the development of sustainable alternatives at an industrial scale for the postharvest protection of berries that can contribute to improving the quality and quantity of fruit production, reducing postharvest losses and enhancing food security [2,107]. However, there are also several challenges associated with the implementation of sustainable alternatives for berries protection on a large scale, including cost, safety, compatibility, scaling up, and regulatory policies (Figure 4). One of the significant challenges in developing sustainable alternatives is the high cost of production. While the use of synthetic fungicides and other chemical treatments is relatively cheap, some sustainable alternatives, such as nanomaterials, can be expensive, and this may lead to profitability reduction [127]. Moreover, the implementation of sustainable methods requires specific knowledge and skills, thereby limiting their widespread application [107].

Another concern is the efficacy of sustainable protection methods against the diverse pathogens that berries encounter during harvesting, storage, and transportation. Moreover, improper hygienic and manufacturing practices promote their contamination with pathogenic bacteria such as *E. coli* and *Salmonella*, requiring customized treatment approaches, making it a complex and time-consuming process [6,66]. Large-scale industrial applications require the development of efficient technologies that can detect and respond to these challenges in real-time. This issue is less relevant in using fungicides and disinfectants because, in most cases, they have activity against several microorganisms [127].

Sustainable alternatives must be safe for consumption to protect human health. It is essential to ensure that the use of nanomaterials and other alternative solutions does not pose any risks to human health. In addition to this, the selected technique should be compatible with the fruit’s requirements during transportation and storage, such as temperature and humidity [6,60]. Currently, most of the alternatives reviewed in this paper were tested on a small scale. There is a need to scale up production to meet the demand for a large quantity of fruits. The challenge is to translate the laboratory concept of a sustainable alternative for the industrial scale. Finally, regulatory issues around the use of natural compounds and nano-based materials in the food industry remain a significant challenge. The implementation of sustainable alternatives at an industrial scale for berry protection is governed by several regulatory frameworks that ensure the use of safe and appropriate substances and technologies. Adherence to these regulations takes time and requires strict compliance, posing a challenge to the widespread adoption of sustainable protection methods [107].

Despite these points, an increase in research interest has led to the development of several sustainable alternative approaches to the postharvest protection of berries, including the use of nanotechnology-based coatings and natural-based solutions. The scientists’ efforts are mainly focused on developing novel technologies and techniques in laboratory-based experiments. The gap between the research and industrial sectors should be reduced and aimed to promote a quick advance in the scale-up of the use of these technologies for berry preservation. Green alternatives for the postharvest protection of berries at an industrial scale are crucial for addressing food security challenges by preserving fruit quality and reducing postharvest losses, which are significant contributors to food waste.

## 8. Conclusions

Berry preservation is crucial for extending its shelf life and maintaining its quality. Traditional methods of preserving berries often involve the use of chemicals and other harmful techniques, which can have negative impacts on the environment and human health. However, several sustainable and eco-friendly postharvest protection strategies can be employed to preserve berries. These alternatives include the application of physical treatments, such as cold storage, modified environment packaging, natural coatings, and so on, as well as the use of natural substances, such as organic acids and essential oils. Additionally, advancements in nanotechnology have led to the development of nanocomposite coatings that can effectively protect berries from spoilage and extend their shelf life. Regarding this, the use of CDs is a promising alternative to developing smart coatings and packaging to enhance the shelf life of berries through agro-waste valorization. These strategies offer promising alternatives to the traditional methods and can contribute to a more sustainable and environmentally friendly approach to berry preservation regarding the quality and safety of berries while minimizing our impact on the environment. The combination of two or more treatments can provide better results. However, it is important to consider that these technologies’ effectiveness strongly depends on the conditions used during the treatment (temperature, concentration, exposure time, etc.). Otherwise, the use of tools based on molecular biology is a promising alternative, of which the main concern is the resistance of the population to consume genetically engineered foods. The further research should be addressed to have a comprehensive understanding of the interaction of these factors and their effect on the microbiological, physicochemical, and sensorial properties of berries. Meanwhile, the joint work of the scientists, industry, and government is the most reliable way to overcome the challenge that implies the implementation of sustainable alternatives for berry preservation. Investing in sustainable postharvest preservation practices can provide a variety of long-term benefits beyond immediate protection. These benefits have far-reaching implications for the environment, the economy, food security, and the overall sustainability of the agricultural systems.

## Figures and Tables

**Table 2:** Edible coatings for berry preservation.

Berry	Coating Composition	Nanoparticle Wall Materials	Bioactive Compound	Method to Obtain the Particles	Coating Technique	Storage Conditions	Significant Result	Reference
Strawberry	Xanthan gum and propylene glycol	Beeswax solid lipid nanoparticles	--	Homogenization	Dipping	4 °C/21 days	Coatings increased the shelf life of strawberries stored in refrigeration.	[98]
	Carboxymethylcellulose	Lactoferrin, chitosan, and gellan solutions.	--	Homogenization	Dipping	25 °C, 50% RH 6 days	Carboxymethylcellulose enhances the adhesion of particles to the fruits.	[99]
	Sodium alginate	--	ZnO suspensions	Ultrasonic homogenization	Dipping	20 days at 1 °C and 95% RH	Higher antioxidant and superoxide dismutase activity, the lowest peroxidase activity, and received the highest-ranked sensory attributes.	[9]
	Particle nanoemulsion	Sodium alginate	Tea tree and cucumber seed oil	Dispersion by stirring	Brushing	25 °C/18 days	Inhibition of microbial growth and delaying fruit maturation, indicating its potential for prolonging the shelf life of fresh food.	[17]
	Sodium alginate	Bagasse cellulose nanocrystals and chitosan nanofibers	Oregano essential oil	High-pressure homogenization.	Dipping	25 °C/9 days	Coating retained desired moisture, respiration rate, stiffness, firmness, and appearance properties of strawberries due to its gas barrier properties, resulting from the entangled matrix structure.	[18]
	Carboxymethylcellulose	Lactoferrin, chitosan and tripolyphosphate (TTP)	–	Ionic cross-linking	Dipping	25 °C, 50% RH for 6 days	Applied to strawberries, the nanoparticles delayed the ripening and degradation of the fruit. Additionally, the antimicrobial properties of lactoferrin and chitosan were intensified by the ionic cross-linking with TPP.	[16]
	Polyvinyl alcohol (PVA)	--	Carbon dots from carob molasses	Hydrothermal process	Dipping	4 °C/12 days	Coating extended shelf life by reducing fungal development and spoilage, as well as moisture loss.	[100]
	Pectin (3%) from orange peels	–	Reuterin and lemon essential oil	Stirring	Dipping	4 °C/31 days	Coatings can avoid fungal spoilage without quality reduction	[101]
	Cellulose nanofibers	--	Carbon dots from glucose and urea	Hydrothermal process/Nitrogen-doped	Dipping	25 °C/2 days	Inhibited fungal growth on the fruit surface and controlled microbial growth.	[102]
Blueberry	Konjac glucomannan/low acyl gellan gum	β-cyclodextrin	Thymol	Spray-drying	Atomization	25 °C/14 days	The combination of heat treatment at 45 °C/60 min and coatings maintained the level of ascorbic acid, total anthocyanin, total acid, and soluble solids and improved the aroma of the coated fruit during the storage.	[103]
	Starch and gelatin	--	Cinnamon essential oil	--	Dipping	4 °C/10 days	Inhibited the growth of molds and yeasts and reduced the ROS level and the activity of superoxide dismutase and catalase by 82, 56, and 63%, respectively, in comparison to uncoated fruits.	[4]
	β-hydroxy-β-methylbutyrate calcium and nanocellulose	--	*Aloe vera*	--	Dipping	4 °C/15 days	Improved the resistance to external forces and reduced the respiration rate, weight loss, and relative electrical conductivity of the fruit, which significantly delayed softening, decomposition, and consumption of total soluble solids and titratable acidity during storage	[104]
	Chitosan		*A. vera*	--	Dipping	5 °C/25 days	Reduced microbiological growth and water loss levels by 50 and 42%, respectively, in comparison to uncoated blueberries. Uncoated fruits showed mold contamination after 2nd day of storage (2.0 ± 0.32 Log CFU/g), whereas coated fruits after the 9th day reached 1.3 ± 0.35 Log CFU/g.	[105]
Grape	Chitosan	--	Lemongrass essential oil	--	Dipping	4 and 25 °C/7 days	Reduced the microbial development on the surface of the fruits and inhibited *Salmonella* growth, maintaining the sensorial properties.	[66]
	Poly(lactic acid) (PLA)	--	*Ocimum basilicum* L. and *Ocimum gratissimum* L. essential oil	--	Nanofibers by solution blow spinning	25 °C/10 days	Reduction between 10 and 12% in comparison to the control and preserved the organoleptic, sensory, and nutritional properties of the fruits.	[67]

**Table 3:** Pros and cons of the main sustainable alternatives applied for berry preservation.

Technique	Advantages	Disadvantages	Reference
Chemical compounds	Inhibition of phytopathogenic fungi. Induction of stress responses. Ethylene oxidation. Inhibition of enzymatic activity. Low cost of implementation at an industrial scale.	High concentrations cause discoloration, texture changes, and chemical burns. Reduction in anthocyanin content. Modification of taste and aroma. Activity affected by environmental conditions and by interaction with food components. Cytotoxic effect at high concentrations in plant cells.	[6,13]
Modified atmosphere packaging	Reduction in physical damage during transportation and storage due to the packaging. Ethylene absorption. Freshness preservation.	Moisture condensation. It does not eliminate the bacteria, and the growth of anaerobic microorganisms can be promoted.	[6]
Low temperature	Decrease in microbial growth rate, reduction in respiration rate and water loss, delaying the ripening and senescence processes.	Temperatures below freezing produce mushy fruits that lose their texture and flavor. Reduction in vitamin C.	[4,108]
Ultraviolet (UV) irradiation	Inhibition of microbial load. Stimulation of the production of anthocyanins and flavonoids, improving the color, taste, and antioxidant properties of berries. Fast and relatively low cost on a large scale.	Excessive exposure to UV light can cause damage to the cellular components of the berries, reducing their quality and shelf life. Poor penetration capacity. High cost. Low consumer acceptance.	[84,85]
Pulsed electric field	Useful at the industrial scale. Maintenance of nutritional value.	High cost of implementation at the industrial scale. Strong conditions can affect vegetable cells, causing softening.	[87,88]
Cold plasma	Changes in the metabolism that extend the shelf life.	Diminution of anthocyanins content. Softening. High cost.	[19,89]
Ionized irradiation	Induction of stress response in the berries, increasing the production of antioxidants and other protective compounds, extending their shelf life.	Reduction in citric acid content in berries. High cost, low consumer acceptance.	[7,8]
Ultrasound	Low cost of implementation. Inhibition of enzymes.	Softening	[93,95]
Edible coatings	Low cost of implementation. Generation of added value products. Increment of the nutritional value. Fully consumed. Enhancement of the organoleptic properties. Carrier of antioxidant and antimicrobial properties. Reduction in weight loss.	Fermentation of the coated foods. Optimization according to the requirements of each fruit. Instability depends on storage conditions (polymers can absorb large amounts of water).	[96,98]

## Data Availability

The data are contained within the article.
